# Congenital Dyserythropoietic Anemia Type I in Association with CDAN1 c.2605 G>A Homozygous Mutation

**DOI:** 10.7759/cureus.115252

**Published:** 2026-08-26

**Authors:** Khalaf H Gargary, Nasir Al-Allawi, Rozhgar Mohammed, Kevi J Ibrahim

**Affiliations:** 1 Pediatrics, College of Medicine, University of Duhok, Duhok, IRQ; 2 Pathology, College of Medicine, University of Duhok, Duhok, IRQ; 3 Hematology, Vajeen Specialist Hospital, Duhok, IRQ; 4 Biology, Science College, Salahaddin University, Erbil, IRQ; 5 Hematology, Maternity Hospital, Duhok, IRQ

**Keywords:** cda1, cdan1 gene, congenital dyserythopoietic anemia, inherited anemia, whole-exome sequencing

## Abstract

Congenital dyserythropoietic anemia type 1 (CDA-1) is a rare autosomal recessive disorder. We report the case of a two-year-old female who presented at three days of age with neonatal jaundice and anemia. She subsequently developed persistent, moderate normochromic anemia with relative reticulocytopenia. Bone marrow aspirate demonstrated erythroid hyperplasia and mild dyserythropoiesis. Whole-exome sequencing (WES) identified a homozygous c.2605G>A (p.V869M) mutation in the *CDAN1* gene, consistent with the diagnosis of CDA-1. Although this variant has been previously documented in a compound heterozygous state, to the best of our knowledge, this report represents its first identification in a homozygous state in CDA-1. This case underscores the utility of genetic testing in diagnosing CDA-1, particularly when morphological changes are subtle.

## Introduction

Congenital dyserythropoietic anemias (CDA) are inherited disorders of erythropoiesis encompassing three classical types (types I, II, and III), transcription-factor-related CDAs, and CDA variants. They are hypoproductive anemias resulting from abnormalities in late erythropoiesis [1]. CDA type I (CDA-1) is a rare autosomal recessive disorder characterized by variable, lifelong, usually macrocytic anemia, relative reticulocytopenia, and distinct morphological abnormalities in late-stage erythroblasts, particularly internuclear bridges. Due to its phenotypic heterogeneity and overlapping features with other inherited anemias, diagnosis of CDA-1 is challenging and requires genetic testing [2,3]. To the best of our knowledge, the current report is the first to describe CDA-1 associated with a homozygous *CDAN1 c.2605G>A* mutation.

## Case presentation

The patient was a two-year-old female with chronic anemia since birth. She first presented at the age of three days with neonatal jaundice and anemia. Her anemia necessitated blood transfusions on two occasions: the first at five days and the other at two months of age. Her parents were not consanguineous, and she was their only living child. Moreover, her mother gave birth to two earlier premature babies, one of whom died at birth, while the other survived for only two weeks. The cause of prematurity was unknown in both babies. On examination, the patient did not have any skeletal abnormalities or dysmorphic features, but she had pallor with a tinge of jaundice. The spleen was not palpable on examination and was not enlarged on ultrasound.

Since her initial presentation, the patient’s hemoglobin levels ranged from 6.4 to 8.9 g/dL, mean corpuscular volume (MCV) from 87.5 to 93.7 fL, and reticulocyte counts from 4% to 5%. At the last visit before her diagnosis, her hemoglobin was 8.9 g/dL, MCV was 89.6 fL, red cell distribution width (RDW) was 23.4%, and reticulocyte count was 4.0% (Table 1).

**Table 1:** Hematological parameters over the patient’s disease course *Three days after the patient's second transfusion; m: months, d: days.

Parameter	Age (in months)						
	2 m	2 m+3d*	3 m	18 m	24 m	30 m	36 m
Hemoglobin (g/dL)	6.4	7.8	7.4	8.8	8.9	9.8	9.5
Normal range (g/dL)	9.4-13.0		11.1-14.1	11.1-14.0	11.0-14.0		
Mean corpuscular volume (fL)	89	89.4	87.5	93.7	89.6	83.3	89.9
Normal range (fL)	87-103		68-84	72-84	75-87		
Mean corpuscular hemoglobin (pg)	32	31.9	27.9	29.0	28.9	27.2	29.3
Normal range (pg)	27-33		24-30	25-29	24-30		
Red cell distribution width (%)	17.9	18.0	20.0	21.2	23.4	18.5	21.0
Normal range (%)	11.0-16.5				11.0-15.0		
Reticulocyte count (%)	-	5.0	-	4.0	4.0	3.3	2.0
Reticulocyte index (%)	-	3.5	-	2.8	2.8	2.6	1.5
Normal range (%)	0.5-3.1			0.5-2.5			

**Figure 1:**
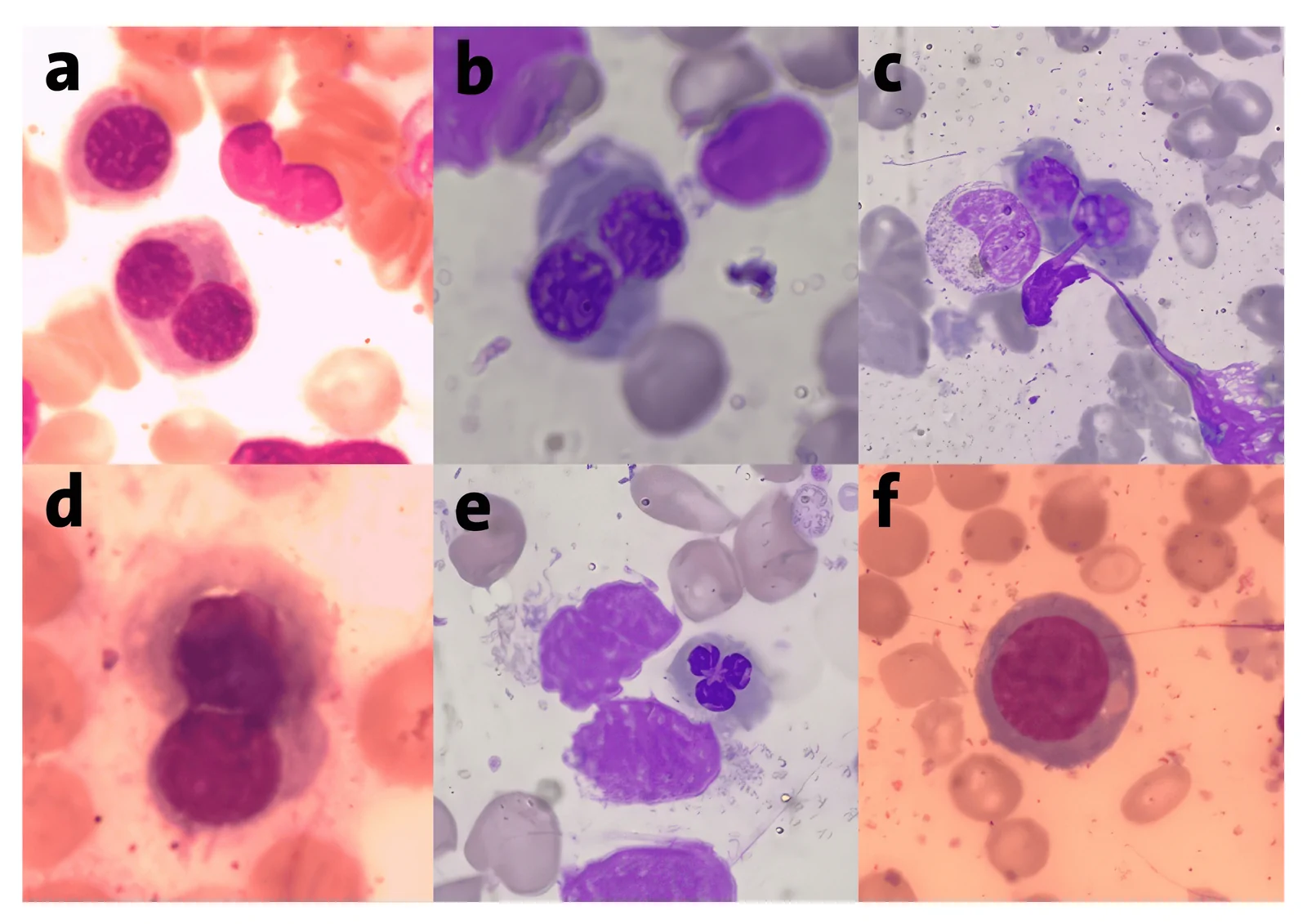
Images from the bone marrow aspirate of the patient documenting some of the dyserythropoietic changes observed. a, b: Binucleated erythroblasts; b-d: Internuclear bridges; e: Cloverleaf nucleus in an erythroblast; f: Megaloblastoid change. (Leishman stain; magnification: x1000)

The peripheral blood picture revealed normochromic red cells with marked anisopoikilocytosis, including oval, teardrop, and macrocytic forms, with occasional nucleated red cells. High-performance liquid chromatography revealed a normal hemoglobin pattern, while the direct Coombs test was negative. Serum bilirubin was 2.2 mg/dL (normal range (NR), 0.2-1.2 mg/dL); serum lactate dehydrogenase (LDH) was 802 U/L (NR, 226-456 U/L); serum ferritin was 276 ng/mL (NR, 12-300 ng/mL); serum vitamin B12 was 746 pg/mL (NR, 200-1515 pg/mL); and serum folate was 10.7 ng/mL (NR, 3.8-16.3 ng/mL). Bone marrow aspiration revealed hyperactive erythropoiesis and megaloblastoid changes, with some nuclear and cytoplasmic dysplasia, including binucleated forms in 3.5%, cloverleaf nuclei, and some internuclear bridges (Figure 1). Myeloid and megakaryocytic precursors were normal. Iron stain showed occasional ringed sideroblasts. CDA-1 was suspected since the patient had two of the four CDA-1 diagnostic criteria, as listed in Table 2 [3].

**Table 2:** Hematological criteria for suspecting the diagnosis of congenital dyserythropoieitc anemia type 1 (CDA-1) Reference: [3]

	Criteria for Suspecting CDA-1
i	Mean corpuscular volume ≥ 90 fL
ii	Hyperactive bone marrow erythropoiesis with dyserythropoiesis indicative of CDA-I
iii	Relative reticulocytopenia, documented by Bone Marrow Responsiveness Index < 120
iv	A tendency towards iron overload (serum ferritin > 300 ng/ml or transferrin saturation ≥ 45%)
Satisfying two of the four criteria should trigger molecular testing to confirm CDA-1	

The two criteria that the patient had were: morphological features in the bone marrow and relative reticulocytopenia (BMRI=91). Accordingly, whole-exome sequencing (WES) was performed on DNA isolated from the patient using the Twist Human Comprehensive Exome (hg38) kit (Twist Bioscience, South San Francisco, CA, USA) on the MGI DNBSEQ-T7 next-generation sequencing platform (MGI Tech Co., Ltd., Shenzhen, China). In silico analysis of the sequencing data was performed using the Genomize SEQ Platform (Genomize, Istanbul, Turkey). The analysis revealed that the patient was homozygous for the mutation *CDAN1*:c.2605 G>A (p.V869M) (NM_138477.4; ClinVar ID: 888272), a mutation that had been associated with CDA-1.

The patient was started on folic acid and monitored over the next 12 months (up to the age of three years). Her clinical course remained unremarkable, and she continued to be transfusion-independent. Her most recent laboratory results showed a hemoglobin level of 9.5 g/dL, MCV of 89.9 fL, a reticulocyte count of 2%, and serum ferritin at 334 ng/mL (Table 1).

## Discussion

In the current case report, the baby was suspected of having CDA-1 because she satisfied two of the four CDA-1 criteria outlined above, namely: morphological features in the bone marrow and relative reticulocytopenia. This led to genetic testing using whole-exome sequencing. The latter revealed that the patient is homozygous for *CDAN1 c.2605 G>A* (p.V869M), a missense mutation located in exon 19 of this gene. This mutation was reported as a Variant of Uncertain Significance (VUS) by the ClinVar database, while it was considered “disease-causing” by the Mutation Taster 2 software [4,5]. According to the ClinVar database, there are more than 700 mutations reported in the *CDAN1* gene, including 67 pathogenic/likely pathogenic, 324 as VUS, while the rest are either benign, likely benign, or of conflicting significance [4]. *CDAN1:c.2605 G>A* was reported once before in a compound heterozygous state in association with CDA-1 [6,7]. Mutations involving *CDAN1* are responsible for nearly three-quarters of those implicated in CDA-1 [3]. The *CDAN1 *gene spans 15 kb and is located on chromosome 15, with 28 exons, directing the synthesis of the protein codanin-1 [6], which is a cell-cycle-regulated protein acting in nucleosome assembly through the formation of an Asf1-H3-H4-importin-4 complex [8].

It is important to note that dyserythropoiesis resembling that seen in CDA-1 could be encountered in other inherited and acquired anemias; therefore, genetic testing is an essential tool for the diagnosis [3,7,9]. Furthermore, in this particular case, the absence of congenital anomalies (encountered in around 10% of CDA-1) and splenomegaly (usually delayed to adolescence or early adulthood in CDA-1) did not offer any additional clues to the diagnosis [3,7]. Moreover, although macrocytic anemia (MCV 100-120 fL) is classical in CDA-1, this was not the case in this patient. This is not unique but has also been noted by earlier studies, such as that of Marra et al. (2024), where 31% of CDA-1 patients had an MCV of 80-90 fL [3].

## Conclusions

This case report, to the best of our knowledge, represents the first documented instance of CDA-I associated with the *CDAN1:c.2605G>A* variant from Iraq. Furthermore, to our knowledge, it is the first report in the literature to describe this specific variant in a homozygous state. It constitutes an important addition to the existing literature, as documenting such cases may pave the way, together with future reports, to upgrade the current variant's classification from VUS.
